# Revealing the diversity of Fusarium micromycetes in agroecosystems of the North Caucasus plains for replenishing the State Collection of Phytopathogenic Microorganisms of the All-Russian Scientif ic Research Institute of a Phytopathology

**DOI:** 10.18699/VJ21.101

**Published:** 2021-12

**Authors:** N.S. Zhemchuzhina, M.I. Kiseleva, Т.М. Kolomiets, I.B. Ablova, А.P. Glinushkin, S.А. Elizarova

**Affiliations:** All-Russian Scientific Research Institute of a Phytopathology (ARSRIP), Moscow region, Russia; All-Russian Scientific Research Institute of a Phytopathology (ARSRIP), Moscow region, Russia; All-Russian Scientific Research Institute of a Phytopathology (ARSRIP), Moscow region, Russia; National Center of Grain named after P.P. Lukyanenko, Krasnodar, Russia; All-Russian Scientific Research Institute of a Phytopathology (ARSRIP), Moscow region, Russia; All-Russian Scientific Research Institute of a Phytopathology (ARSRIP), Moscow region, Russia

**Keywords:** microorganism collections, micromycetes, genetic diversity, winter wheat, plant pathogens, Fusarium, коллекции микроорганизмов, микромицеты, генетическое разнообразие, озимая пшеница, фитопатогены, Fusarium

## Abstract

In order to prevent crop yield losses from the most dangerous and economically important pathogenic organisms, it is necessary not only to monitor the virulence gene pool, but also to study the nature of pathogen variability and determine the potential for the emergence of new genes and races. This requires centralized collections of fungal cultures characterized by a set of stable strains to provide for phytopathological, immunological, breeding, genetic, toxicological, parasitological and other studies. The State Collection of Phytopathogenic Microorganisms of the ARSRIP is the State Depository of Phytopathogenic microorganisms that are non-pathogenic to humans or farmed animals. Currently, it has more than 4,500 accessions of plant pathogenic strains of fungi, oomycetes, bacteria, viruses, phytoplasmas, and the collection is updated annually. For this purpose, the study of the inter- and intraspecific genetic diversity of genus Fusarium was carried out in agricultural systems of the Krasnodar Territory. In 2020, the State Collection of Phytopathogenic Microorganisms was supplemented with 13 strains of Fusarium fungi isolated from tissues of winter wheat plants collected in several locations of the Krasnodar region. The complex of Fusarium fungi revealed on winter wheat usually included Fusarium oxysporum, F. culmorum, F. lolii, F. graminearum, F. fujikuroi, F. sporotrichioides, etc. The effect of the preceding crop on the frequency of Fusarium species isolated from winter wheat was observed. After series cloning of collected isolates, 21 strains of different fungal species characterized by stable morphology traits and known pathogenic and phytotoxic properties were selected for collection replenishment. Significant differences in pathogenic activity were revealed between fungi belonging to either the same or different species; the manifestation of this activity varied from the absence of any effect of spore suspensions on seedling development to a complete inhibition of their growth. The phytotoxic activity towards wheat seedlings varied from medium to high. Species possessing a high intensity of phytotoxic activities are the most dangerous for wheat, since they promote accumulation of dangerous phytotoxins in plant tissues.

## Abstract (ru)

С целью предотвращения потерь урожая сельскохозяйственных культур от наиболее опасных и экономически значимых патогенных организмов нужно не только проводить контроль генофонда вирулентно- сти, но и изучать характер изменчивости патогенов, определять потенциальную возможность появления новых генов и рас. Для этого необходимы централизованные коллекции живых культур, характеризующиеся набором стабильных штаммов для обеспечения фитопатологических, иммунологических, селекционных, генетических, токсикологических, паразитологических и других исследований. Государственная коллекция фитопатогенных микроорганизмов Всероссийского научно-исследовательского института фитопатологии – государственный де- позитарий фитопатогенных микроорганизмов, непатогенных для человека и сельскохозяйственных животных. В настоящее время она насчитывает более 4500 единиц хранения патогенных для растений штаммов – грибов, оомицетов, бактерий, вирусов, фитоплазм – и ежегодно пополняется. С этой целью было проведено изучение межвидового и внутривидового генетического разнообразия фузариевых грибов в агроэкосистемах Краснодар- ского края. В 2020 г. Государственная коллекция фитопатогенных микроорганизмов была пополнена штамма- ми 13 видов грибов рода Fusarium, выделенными из тканей растений озимой пшеницы из нескольких районов Краснодарского края. В комплексе фузариевых грибов озимой пшеницы наиболее часто выявляли Fusarium oxysporum, F. culmorum, F. lolii, F. graminearum, F. fujikuroi, F. sporotrichioides и др. Отмечено влияние предшественника озимой пшеницы на частоту выделяемых видов грибов рода Fusarium. После серии моноконидиальных клони- рований изолятов в коллекцию был отобран 21 штамм грибов разных видов со стабильными морфолого-куль- туральными признаками и известными патогенными и фитотоксичными свойствами. Патогенная активность у грибов как между видами Fusarium, так и в пределах одного вида существенно различалась: от отсутствия при- знаков влияния споровых суспензий на развитие проростков до полного их угнетения. Фитотоксическая ак- тивность в отношении проростков пшеницы варьировала от средней до высокой. Наибольшую опасность для проростков пшеницы представляют виды, обладающие высокой интенсивностью проявления фитотоксической активности, так как они способствуют накоплению опасных фитотоксинов в тканях растений.

## Introduction

To successfully solve the problems of food security of the country, it is necessary to create varieties resistant to particularly dangerous diseases. In order to prevent crop yield losses from the most dangerous and economically significant pathogenic organisms, it is necessary not only to monitor the virulence gene pool, but also to study the nature of pathogen variability, determine the potential for the appearance of new genes and possibly dangerous races in different fungi populations. This requires centralized collections of cultures characterized by a set of stable properties to provide for phytopathological, immunological, breeding, genetic, toxicological, parasitological and other studies. Such collections of phytopathogenic organisms have been created and are successfully functioning in most developed countries of the world.

The State Collection of Phytopathogenic Microorganisms of the All-Russian Scientific Research Institute of a Phytopathology (ARSRIP) is the main gene pool of races, biotypes, pathotypes of phytopathogenic fungi, bacteria and viruses distributed over the vast territory of the Russian Federation. This is the first such gene pool created in Russia. Until recently, there were only scattered working collections of individual species of phytopathogenic microorganisms in various institutions and departments of the institute. Collection of phytopathogenic microorganisms of ARSRIP by the decree of the Government of the Russian Federation “On measures for the conservation and rational use of collections of microorganisms” dated 24.06.1996. No. 725-47c was given the name “State Collection of Phytopathogenic Microorganisms and Varieties-Identifiers (Differentiators) of Pathogenic Strains of Microorganisms” and the status of the State Depository of phytopathogenic microorganisms that are not pathogenic to humans and farm animals was determined. Currently, it has more than 4,500 storage units of plant pathogenic strains – fungi, oomycetes, bacteria, viruses, phytoplasmas – and is updated annually. For this purpose, the study of inter- and intraspecific diversity of Fusarium fungi in agricultural systems of the Krasnodar Territory was carried out.

According to the literature, facultative parasites from the genus Fusarium are often observed on winter wheat. These micromycetes are well adapted to changing external environmental factors, which ensures their survival in a wide range of weather conditions, and therefore are distributed almost everywhere where winter wheat is cultivated (Rukavitsina, 2008; Chulkina et al., 2009; Toropova et al., 2013). Monitoring the structure and localization of Fusarium populations in wheat ecosystems is of great practical importance not only for selecting disease-resistant varieties, but also for increasing the effectiveness of protective measures and improving the environmental situation of agricultural crops.

Recently in the southern regions of Russia, where winter wheat is widely cultivated, there has been an increase in diseases caused by fungi of the genus Fusarium (Zhalieva, 2010). It is known that 28 species of fungi of this genus parasitize wheat. The species Fusarium graminearum, F. poae, F. sporotrichioides, F. tricinctum, F. nivale prevail on wheat in the North Caucasus. As a rule, they are observed as pathogens of root rot, causing the weakening and death of seedlings, reducing the productivity potential of affected adult plants

In some years, Fusarium head is widely spread, causing significant damage to grain production. On vegetative and generative organs of plants, the species composition of fungi can be ambiguous depending on weather conditions, the stability of cultivated varieties, wheat precursors, agricultural technology and many other factors (Chulkina et al., 2009; Zhalieva, 2010).

Assessment of the diversity of morphological features of Fusarium spp., identification of the amplitude of their variability, including the level of pathogenicity and phytotoxicity, is important for the selection and replenishment of the collection with micromycete strains (Booth, 1971; Thrane et al., 2004; Kolomiets et al., 2018).

The need to preserve the material of Fusarium spp. strains and isolates and the constant selection of samples to replenish the collection is explained by the relevance of conducting scientific research for the development of methods of biological protection, to study the dynamics of the development and settlement of fungi, to assess their pathogenic and toxic activity on host plants. The collection material is also necessary for the evaluation and selection of wheat samples for breeding for resistance to disease (Dubovoy et al., 2016; Zhemchuzhina, Elizarova, 2019).

In this regard, the main tasks of a collection of unique Fusarium spp. isolates is not only to preserve the viability of spores and their genetic stability according to morphological and cultural characteristics for a long time, but also to replenish the funds with new isolates with a different spectrum of pathogenic properties, as well as to expand the range of geographical territories for selecting isolates (Gagkaeva, Levitin, 2005; Gagkaeva et al., 2008).

To fulfill these tasks, samples of infected plants received annually from various regions of the country are mycology studied, and the most pathogenic and phytotoxic samples are selected for the collection. Strains and isolates from the genus Fusarium have an important place in the collection, which serves, as has already been mentioned, to maintain the strains of these microorganisms in a viable state, preserve their pathogenic properties and provide infectious material for phytopathological, immunological, breeding, genetic and toxicological studies (Kolomiets, Zhemchuzhina, 2018).

The purpose of these studies was to assess the species and intraspecific diversity of micromycetes of the genus Fusarium in wheat crops in agroecosystems of the Krasnodar Territory for the selection and replenishment in 2020 of the State Collection of Phytopathogenic Microorganisms (SCPM) with strains and isolates of fungi isolated from the roots and leaves of vegetating winter wheat plants in several districts of the Krasnodar Territory.

## Materials and methods

The research material was plants of zoned varieties of winter wheat with signs of fungal infections on the leaves and roots. The samples were selected in the second decade of May 2019 from winter wheat crops for different predecessors in the Pavlovsky, Korenovsky, Ust-Labinsky, Kanevsky and Primorsko- Akhtarsky districts of the Krasnodar Territory. The samples contained 10–20 wheat plants in the earing-grain formation phase. All the works were performed using the equipment of the Center of Collective Usage SCPM ARSRIP (http://www. vniif.ru/vniif/page/ckp-gkmf/1373).

Fungi were isolated from the affected plants using 2 % potato-glucose and potato-carrot agar. Fungi from wheat samples were isolated according to the standard method (Bilai, 1977; Bilai, Ellanskaya, 1982). The infected plants of each sample washed with tap water were cut into fragments of 5–10 mm in size, sterilized in 50 % alcohol for 20–30 seconds and, under aseptic conditions, laid out on the surface of 2 % potato-glucose agar in Petri dishes (4–6 fragments each). Each sample was represented by at least 150–00 fragments of the affected tissue. Petri dishes were placed in a thermostat with a temperature of 22–24 °С.

Observation of the development of fungi was carried out daily. As the fungal colonies grew, a piece of mycelium was sifted onto the nutrient medium in the center of the Petri dish. Fungal cultures were examined under a microscope. The species of fungi of the genus Fusarium were determined by the main morphological features of colonies and spores: by the growth rate, the color of the mycelium and its structure, pigmentation; by the shape and size of the apical and basal cells of macroconidia, by the presence of microconidia. 300 conidia were estimated for an average size of macroconidia. For determining the species of the fungus (Gerlach, Nirenberg, 1982; Leslie, Summerell, 2006; Dictionary..., 2008; Watanabe, 2010) were used as reference literature.

The assessment of the degree of sporulation was carried out on 14-day colonies of the fungus. At the same time, the results of sporulation were determined by the average value of the number of spores per cup when flushing from 10 Petri dishes of one morphotype. The sporulating ability of fungal colonies was determined by the standard method of counting spores in the Goryaev chamber (Bilai, 1977).

Series of monospore cloning of micromycete isolates were carried out according to the generally accepted method for the selection of strains of Fusarium fungi stable by morphological and cultural characteristics (Bilai, 1977; Bilai, Ellanskaya, 1982).

Fungal isolates isolated from the affected wheat samples were stored in a refrigerator at a temperature of 7–10 °С in biological test tubes on the nutrient medium – potato-glucose agar (Bilai, Ellanskaya, 1982).

The pathogenic and toxic properties of the strains were studied using the method of bioassay on seeds. The pathogenicity of spore suspensions and phytotoxicity of filtrates of culture fluids (FCF) of fungi of the genus Fusarium were tested on wheat seeds (Mironovskaya 808 variety). The degree of pathogenicity and toxicity of the strains was judged by the effect of suspensions of conidia and FCF on the germination of wheat seeds, the development of sprouts and primary roots of seedlings. However, the main indicator was considered the length of the roots.

The degree of pathogenicity and toxicity was determined on the 5th day from the beginning of seed germination. If the length of seedlings and roots (in mm) in the experimental version was 0–30 % of the control length, this indicated strong pathogenic (P) and strong toxic (T) activity of the fungus; 31–50 % – moderate pathogenicity (MP) and moderate toxicity (MT); 51–70 % – weak pathogenicity (WP) and weak toxicity (WT); 71–100 % – non-pathogenic (NP) and non-toxic (NT) properties of the isolate. The length of the sprouts and primary roots of seeds sprouted in water was considered as control and was taken as 100 %.

## Results and discussion

During the mycological studies of experimental samples it was noted that fungi of the genus Fusarium had the same symptoms on plant organs, but the pieces of tissue of different organs, washed and flamed over the fire, decomposed into wet chambers, formed a characteristic mycelium and conidia for 3–5 days, which made it possible to identify the type of micromycete. The study of the main micro- and macromorphological features of fungi in culture by the presence, shape and size of macroconidia and microconidia (if present), the growth rate of the colony, the color and structure of the mycelium, carried out on more than 400 isolates of fungi, allowed us to identify the following 13 species of the genus Fusarium: F. oxysporum Schlecht., F. culmorum (Sm.) Sacc., F. lolii (Wm. G. Sm.) Sacc., F. graminearum Schwabe, F. moniliforme J. Sheld. (syn. F. fujikuroi Nirenberg), F. sporotrichioides Swerb., F. avenaceum (Fr.) Sacc., F. poae (Peck) Wollenw., F. sambucinum Fuckel (syn. F. roseum Link), F. acuminatum Ellis & Everh., F. equiseti (Corda) Sacc. (syn. F. gibbosum Appel & Wollenw.), F. chlamydosporum Wollenw. & Reinking, F. solani (Mart.) Sacc. (Table 1).

**Table 1.**
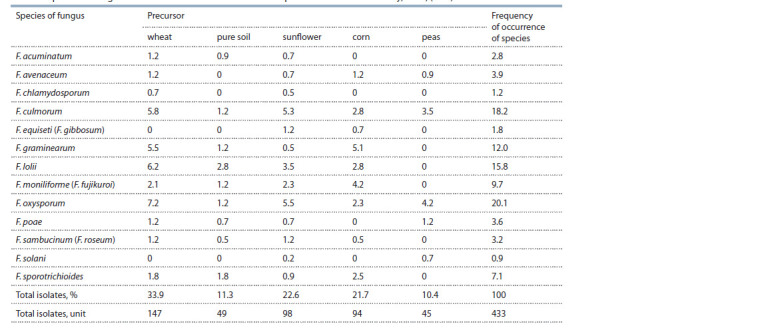
Species of the genus Fusarium found on winter wheat crops in the Krasnodar Territory, 2019, (in %)

In the complex of Fusarium fungi of winter wheat in the Krasnodar Territory in 2019, isolates of F. oxysporum (Schlecht.) Snyd. et Hans. were detected most often (20.1 %). The fungus was recorded on winter wheat crops in the Ust- Labinsk, Pavlovsky, Korenovsky, Primorsko-Akhtarsky districts. Undoubtedly, the culture preceding winter wheat had an impact on the frequency of occurrence of F. oxysporum. Thus, the share of those isolates according to the wheat precursor was 7.2 %, sunflower – 5.5, peas – 4.2, corn –1.2 %. Often, cultures of F. avenaceum, F. acuminatum, F. sambucinum were noted together with this fungus, as well as Alternaria spp., bacteria, etc.

The second place in frequency of occurrence was occupied by F. culmorum (Sm.) Sacc. Isolates of the fungus were obtained from samples of the affected roots and the basal part of the stem of winter wheat in almost all the surveyed areas. The share of this pathogen in the complex of fungi of the genus Fusarium was 18.2 %. Often, isolates of F. culmorum were found in samples of winter wheat, the precursors of which were wheat, sunflower and peas

F. lolii (Wm. G. Sm.) Sacc. (teleomorph Gibberella cyanea (Sollm.) Wr., syn. F. heterosporum Nees.), as a rule, were isolated from the highly rot-damaged and dried roots of winter wheat, the precursors of which were wheat (6.2 %) and sunflower (3.5 %).

Isolates of F. graminearum Schwabe (teleomorph G. zeae (Schwein.) Petch.) were discovered on the roots and basal stems of winter wheat in most areas of infectious material, and the fungus was isolated more frequently if predecessors were wheat and corn (5.1 and 5 %, respectively).

Isolates of F. moniliforme J. Sheld. (teleomorph G. moniliformis Wineland; syn. F. fujikuroi Nirenberg), the causative agent of pink mold and root rot of cereals, were found on the leaves, stems and roots of winter wheat in the Primorsko- Akhtarsky, Pavlovsky and Kanevsky districts. More often, the fungus was isolated from wheat, the precursors of which were corn (4.2 %), sunflower (2.3 %) and wheat (2.1 %).

Isolates of F. sporotrichioides Sherb. were isolated from the affected roots, root neck and stems of winter wheat (7.1 %) from the Pavlovsky and Korenovsky districts.

Isolates of F. avenaceum (Fr.) Sacc. (teleomorph: G. avenacea Cook) were found on leaves, ground parts of stems and roots of winter wheat from the Pavlovsky and Korenovsky districts. In the complex of fungi from the genus Fusarium isolated from winter wheat samples, the frequency of occurrence of F. avenaceum was 3.9 %. The fungus was observed in samples of winter wheat, the previous crops of which were wheat and corn.

The isolates of F. poae (Peck) Wollenw. were found with low frequency (3.6 %) in the Pavlovsky district. More often, isolates of this type were noted on samples of wheat, the precursors of which were wheat and peas

It should be noted that in some cases two or more phytopathogens from the genus Fusarium were isolated from one sample of the affected winter wheat tissue. Such isolates of F. acuminatum Ellis & Verh. (telemorph G. acuminate Wr.) were isolated together with F. oxysporum more than half of the cases.

A similar pattern was noted for F. sambucinum Fuckel (teleomorph G. pulicaris (Fr.) Sacc.). This fungus, regardless of the location on the plant (leaf, stem, root), was always isolated together with F. oxysporum, while it was often accompanied by a bacterial infection.

F. acuminatum isolates were found in the Ust-Labinsk and Pavlovsky districts with low frequency (2.8 %) in the affected roots of winter wheat, the precursors of which were wheat, steam and sunflower.

Isolates of F. equiseti (Corda) Sacc. (teleomorph G. intracans Wollenw.; syn. F. gibbosum Appel & Wollenw. Emend Bilai) were detected mainly on browned winter wheat stalks in the Ust-Labinsk and Korenovsky districts.

The proportion of isolates of F. chlamydosporum Wollenw. &Reinking in the complex of Fusarium fungi did not exceed 1.2 %. The fungus was isolated from the roots of two wheat samples from the Korenovsky district. Along with F. chlamydosporum, saprophytic and pathogenic fungal species were abundantly isolated from the same roots.

Several isolates of F. solani (Mart.) Sacc. (teleomorph Nectria haematococca Berk. & Broome) were found in the Pavlovsky district on the roots of wheat.

As it was noted earlier, based on the study of morphological features, the obtained isolates of fungi of the genus Fusarium are assigned to 13 taxonomic groups. After a series of monoconidial cloning of isolates, strains of fungi of different species with stable morphological and cultural characteristics were selected for the collection. When selecting fungi cultures for the collection, special attention was paid to the macro- and micromorphological features characteristic of each species.

The Fusarium spp. strains differed in the morphology, in the size and shape of macro- and microconidia, and in the sporulation of colonies. Differences between strains within the same species were often noted only when studying macromorphological features – the color and structure of the mycelium, sporulation. When analyzing the data of micromorphological features, i. e., the shape and size of conidia, the method of their formation, the differences between the strains of the same species were minimal.

21 strains of Fusarium spp. identified on winter wheat crops of the North Caucasus in 2019 were transferred to the collection (Table 2).

**Table 2.**
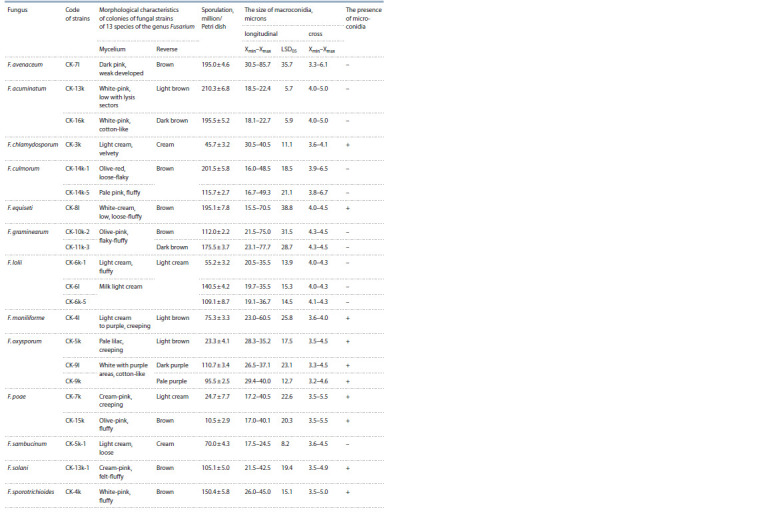
Characteristics of fungal strains of 13 species of the genus Fusarium selected in the SCPM ARSRIP, according to macro- and micromorphological properties

The obtained biological material of Fusarium spp. was studied by the degree of pathogenic and phytotoxic severity. The results of the influence of spore suspensions and metabolites of filtrates of culture fluids of 21 strains of fungi from the genus Fusarium on the development of wheat seedlings in cultivar Mironovskaya 808 (seed germination, sprout and root length) were shown in Table 3.

**Table 3.**
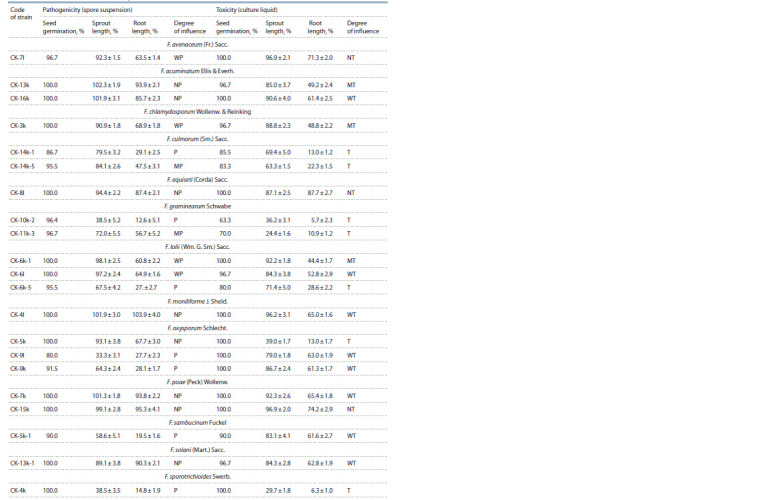
Characteristics of Fusarium fungal strains by pathogenicity of spore suspensions and phytotoxicity of culture liquid on wheat seedlings of cultivar Mironovskaya 808 (in % of control) Notе. NP/NT is non-pathogenic/non-toxic; WP/WT – weakly pathogenic/weakly toxic; MP/MT – moderately pathogenic/moderately toxic; P/T – pathogenic/toxic.

It was shown that the strains of fungi within the same species differed in the pathogenic or phytotoxic degrees. The strains of F. oxysporum (СK-5k, СK-9l, СK-9k) and F. lolii (СK-6k-1, СK-6l, СK-6k-5) had a wide intraspecific diversity according to these characteristics. Among them, there were different categories – from pathogenic/toxic to non-pathogenic/ slightly toxic.

Phytotoxic and pathogenic isolates of F. culmorum and F. graminearum suppressed the development of seedlings of cv. Mironovskaya 808 to a wide extent.

Isolates of F. acuminatum (CK-13k, CK-16k) were found to be non-pathogenic to the seedlings of the tester variety, but had weak and moderate phytotoxicity.

Isolates of F. avenaceum (CK-7l), F. equiseti (CK-8l), F. poae (CK-7k, CK-6k-1) and F. chlamydosporum (CK-3k) and others were characterized by very weak pathogenic and phytotoxic properties.

It was found that spore suspensions of fungal isolates had little effect on seed germination (80–100 %), but subsequently affected the development of seedlings: pathogenic isolates of fungi inhibited their growth (up to 33.3 % strain of F. oxysporum CK-9l) or non-pathogenic ones stimulated it (up to 102.3 % strain of F. acuminatum CK-13k). A stronger effect of spore suspensions on the growth and development of primary roots was noted (12.6–95.3 %).

Seeds’ treatment of filtrates of culture fluids of Fusarium strains had a weak effect on their germination (63.3–100 %), although in the future the intensity of development of seed seedlings significantly slowed down. The length of seedlings under the action of filtrates of fungal culture fluids compared to the control was 24.4–96.9 %. The average length of the primary roots was 5.7–74.2 %, which allowed the isolates to be grouped according to the degree of toxicity.

The obtained results indicate that the Krasnodar populations of Fusarium differ by morphological, pathogenic and phytotoxic characteristics

## Conclusion

The influence of the precursor of winter wheat, from which experimental samples were taken, on the frequency of isolated of the genus Fusarium was noted. It is shown that the pathogenic activity of fungi both between Fusarium species and within the same species differs significantly: from the absence of signs of the influence of spore suspensions on the development of seedlings to their complete suppression. Phytotoxic activity against wheat seedlings varied from medium to high. The greatest danger for wheat seedlings is represented by species with a high intensity of phytotoxic activity associated with the fact that they contribute to the accumulation of dangerous toxins in plant tissues.

Based on the results of the data obtained, the strains of 13 species of the genus Fusarium from the agroecosystems of the lowland part of the North Caucasus were selected and placed in the collection. All strains are characterized by morphological, pathogenic and phytotoxic properties.

## Conflict of interest

The authors declare no conflict of interest.
